# Evolutionary History of Chordate *PAX* Genes: Dynamics of Change in a Complex Gene Family

**DOI:** 10.1371/journal.pone.0073560

**Published:** 2013-09-02

**Authors:** Vanessa Rodrigues Paixão-Côrtes, Francisco Mauro Salzano, Maria Cátira Bortolini

**Affiliations:** Departamento de Genética and Programa de Pós-Graduação em Genética e Biologia Molecular, Instituto de Biociências, Universidade Federal do Rio Grande do Sul, Porto Alegre, Rio Grande do Sul, Brazil; University of Lausanne, Switzerland

## Abstract

Paired box (*PAX*) genes are transcription factors that play important roles in embryonic development. Although the *PAX* gene family occurs in animals only, it is widely distributed. Among the vertebrates, its 9 genes appear to be the product of complete duplication of an original set of 4 genes, followed by an additional partial duplication. Although some studies of *PAX* genes have been conducted, no comprehensive survey of these genes across the entire taxonomic unit has yet been attempted. In this study, we conducted a detailed comparison of *PAX* sequences from 188 chordates, which revealed restricted variation. The absence of *PAX4* and *PAX8* among some species of reptiles and birds was notable; however, all 9 genes were present in all 74 mammalian genomes investigated. A search for signatures of selection indicated that all genes are subject to purifying selection, with a possible constraint relaxation in *PAX4*, *PAX7*, and *PAX8*. This result indicates asymmetric evolution of *PAX* family genes, which can be associated with the emergence of adaptive novelties in the chordate evolutionary trajectory.

## Introduction

Paired box (*PAX*) genes are transcription factors that play key roles in several aspects of embryonic development and organogenesis [1]–[3]. Although the *PAX* family is specific to the animal lineage, the evolutionary history of these genes remains uncertain. A unique *PAX* gene (*TriPaxB*) has been isolated from *Trichoplax adhaerens* (Placozoa), the most morphologically simple species of all non-parasitic multicellular metazoan animals. The TriPaxB protein contains the characteristic DNA-binding domain of the *PAX* family, the paired domain (PD) of 128 amino acids, an octapeptide motif (OC), and a paired-type homeobox DNA-binding domain (HD) [1], [4].

Four *PAX* genes (*PAX1/9, PAX2/5/8, PAX3/7,* and *PAX4/6*) have been found in the basal chordates, amphioxus (*e.g. Brachiostoma floridae*) and tunicates (*e.g. Ciona intestinalis*) [5]–[7]. Phylogenetic analyses indicated that a single *PAX* gene of each subfamily was present in the ancestral chordate and gave rise to the amphioxus *PAX.* Afterwards, a plus round of whole genome duplications, gave origin to the multiple vertebrate *PAX* subfamily copies [4]–[8]. Ohno [9], suggested that the early vertebrate lineage underwent one (1 R) or more (≥2 R) whole genome duplications (WGDs). These processes were considered to provide additional possibilities for diversified evolution and/or speciation. Rapid and widespread evolutionary changes could lead to macroevolutionary emergent properties, since WGD products are able to evolve and reach a greater level of interaction and complexity than would otherwise be possible through cumulative single gene duplications [10]–[15]. The second round of whole genome duplication most likely occurred after the divergence of invertebrate chordate lineages from the ancestral vertebrate, although there is controversy about the exact branch at which the phenomenon occurred [11], [16]–[18].

After these 2 major duplication events occurred, probably 8 *PAX* genes emerged. Another partial duplication occurred subsequently, resulting in the 9* PAX* genes currently found in mammals (subfamilies: (1) *PAX1* and *PAX9;* (2) *PAX2, PAX5*, and *PAX8;* (3) *PAX3* and *PAX7;* (4) *PAX4* and *PAX6* [1], [6]). An alternative scenario would be that more *PAX* genes would have arisen after 2 WGRD and then lost during the vertebrate evolution history [19], [20].

In vertebrates, as well as in other chordates, *PAX* genes are notably expressed during development. They are also known to play an important role in mature life stages, based on observations of organ/tissue-specific signals (Table S1; [21]). For instance, PAX3 and PAX7 proteins are found in adult cells of the vertebrate muscle tissue [22]. Analogously, in amphioxus, the *PAX3/7* gene is most highly expressed in adult muscle [7]. These observations, along with other similar findings, indicate that a *PAX*-derived gene can maintain similar roles to those present in the putative ancestor. Nonetheless, various novel roles for *PAX* genes have also emerged during the evolutionary history of vertebrates: they were co-opted for new regulatory networks, diverged, and subsequently gained new functions [5], [7], [19]–[27].

Although the presence of *PAX* genes has been investigated in a variety of organisms [6], [19]–[23], [26], a broad survey of chordate *PAX* genes has yet to be conducted. Therefore, the aim of the present study was to address questions regarding the occurrence and evolution of the *PAX* family in chordates. We used publicly available sequences to evaluate: (1) the presence/absence of *PAX* genes in 188 organisms, and (2) the evolutionary rates and properties of *PAX* genes in chordates. Results of this analysis will contribute to a greater understanding of the mechanisms of change in complex gene families.

## Results and Discussion

### Search and Identification for Vertebrate *PAX* Genes

**Figure 1:**
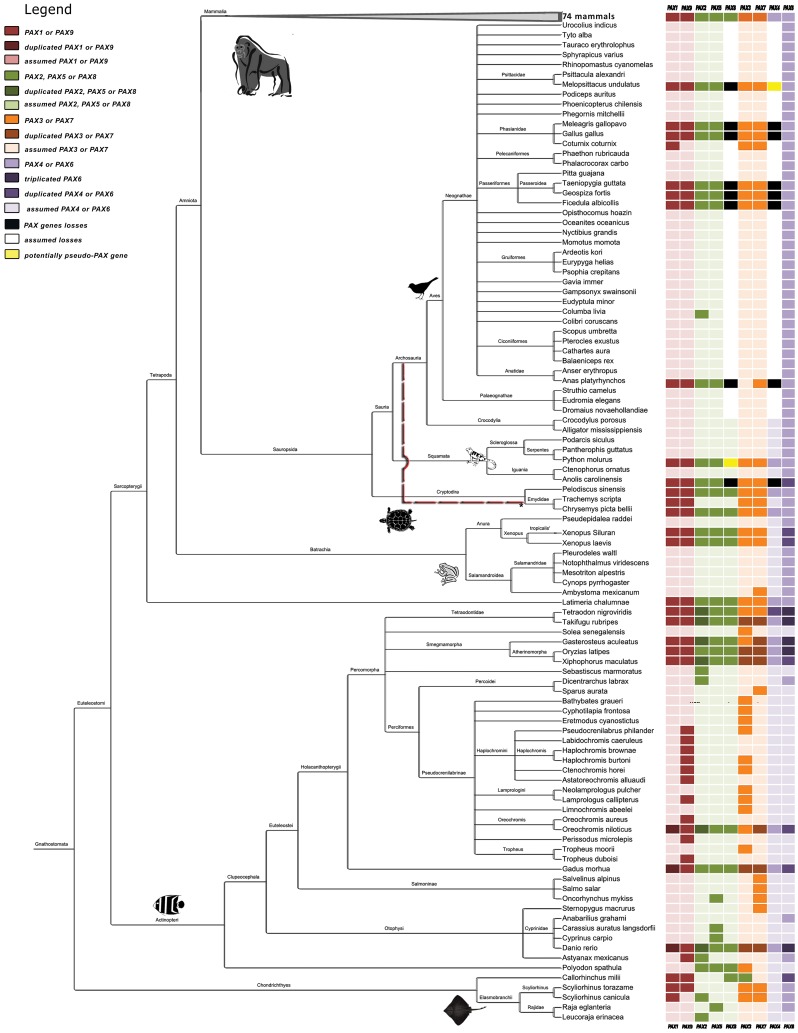
Phylogenetic tree for the 175 jawed vertebrate species considered in this study. Sequences were obtained from the NCBI Taxonomy Browser and edited with Figtree and hitmap for the presence of *PAX* genes. The dashed red line indicates the relationship suggested by Crawford et al [46].

We characterized chordate *PAX* genes using sequences that were available in the Ensembl, UCSC, NCBI, and UniProt databases [28]–[31]. A total of 188 species were evaluated, including vertebrates (175 jawed and 4 jawless), urochordates (6 tunicates), and cephalochordates (3 amphioxus) [32]; see details in Tables S2 and S3 and Figure 1.

### Basal Chordates

We retrieved 4 *PAX* genes in cephalochordates (*Branchiostoma belcheri, B. floridae*, and *B. lanceolatum*), which is the most basal chordate subphylum. In tunicates, the most basal animals belonging to the Olfactores clade, we recovered 5 *PAX* genes. Three of the genes (*PAX1/9, PAX 3/7,* and *PAX4/6*) could be considered equivalent to the ancestral vertebrate *PAX* types, while the others (*PAX2/5/8a* and *PAX2/5/8b* in *Branchiostoma lanceolatum*) were derived [5]–[7], [33].

### Jawless Vertebrates

Jawless vertebrates were represented in our study by 1 hagfish species (*Eptatretus burger*) and 3 lamprey species (*Lampetra fluviatilis, Lethenteron camtschaticum*, and *Petromyzon marinus).* Together, they form a sister group of the gnathostome vertebrates, making them a good model to investigate ancestral vertebrate characteristics. The hidden Markov models (HMMER) search recovered 3 *PAX* genes for the hagfish and 4 for all lampreys. In *Petromyzon marinus*, we recovered 2 *PAX* genes (*PAX1/9* and *PAX1/9b*), and found 2 segments of 158 bp and 144 bp, respectively, showing 88% of identity with the *PAX3* and *PAX7* genes. Interestingly, *in vitro* studies identified *PAX1/9* and *PAX1/9b* in this species [33], as well as the *PAX7, PAX2* [34], *PAX6* [33]–[37], *PAX3/PAX7* genes [36]. This *in vitro* information, which was confirmed by our genome data, suggests that *Petromyzon marinus* contains genes corresponding to the 4 ancestral *PAX* genes in addition to a second copy of the *PAX1/9* and *PAX3/7* type. This suggests that a duplication of the *PAX1/9* and *PAX3/7* genes occurred in the lamprey or jawless lineage, although the possibility of an ancient genome duplication event (before the split between jawless and jawed vertebrates) with subsequent lineage-specific modifications cannot be discarded [12]. The difference between the numbers of *PAX* genes found in the basal chordates, tunicates (4 or 5) and lampreys (6) and the basal jawed vertebrates (9) can be associated with the emergence of adaptive novelties at the tunicate/vertebrate and agnathan/gnathostome transitions.

### Jawed Vertebrates

We found 6 *PAX* genes in the most basal taxon of this group, the Chondrichthyes (2 skates, 2 sharks, and 1 chimaera). The chimaera species (*Callorhinchus milii;* elephant shark), for which the draft genome is already available, contained all 6 genes (1, 9, 8, 3, and 2 copies of 6; Figure 1 and Table S3). *PAX4* was not retrieved in any search (Genomes, HMMER protein, and BLAT/BLAST; Table S3). However, the absence of *PAX4* should be interpreted with caution since the elephant shark genome has low coverage (1.4*x*) and the sequence databases are biased toward the most popular/known genes. The duplicate *PAX6* (named *PAX6.2*) was recently discovered, and based on experimental work and in the conservation of coding and noncoding elements, the authors suggested that although an ancient duplication event occurred in a gnathostome ancestor, the additional copy was independently lost in mammals and birds [38].

All of the expected 9 *PAX* genes were found in 37 species of ray-finned bony fishes. Considering only the 8 ray-finned bony fishes for which complete genomes are available (class Actinopterygii; Table S3), additional duplicate or triplicate copies were found in 7 of the 9 *PAX* genes (exception: *PAX5* and *PAX8*; Figure 1). This situation was probably a consequence of whole genome duplication [39], [40], which occurred in the early evolution of teleost fishes approximately 320–350 million years ago (3 RWGD hypothesis; [41]).

*Latimeria chalumnae* (coelacanth), a lobe-finned fish, presents all 9 *PAX* genes, suggesting that the ancestor that gave rise to the tetrapod lineage contained all members of the *PAX* family.

The frog species *Xenopus tropicalis* and *Xenopus laevis* also presented 9 *PAX* genes. However, we found duplicated copies of *PAX2* and *PAX6* in *X. laevis* and *X. tropicalis*, respectively. The presence of the additional *PAX2* copy in *X. laevis* could be the result to the fact that this species experienced a recent and specific polyploidization event approximately 40 million years ago. Approximately 32–47% of duplicated genes were observed in its whole genome [15], [39], [42]. An alternative is that *PAX2* could have been duplicated through local gene duplication. The duplication of *PAX6* in *X. tropicalis* has been reported in a previous study (*PAX6.2* [38]). For the other 6 species of amphibians, we only retrieved *PAX6* and *PAX7* sequences (6 hits and 1 hit, respectively). For all amphibian species studied here, it was not possible to localize *PAX4*, corroborating a recent paper that proposed that *X. tropicalis* lost *PAX4* [43]. These data suggest that the absence of *PAX4* could be a general characteristic of amphibian taxa.

The analysis of the entire *PAX* family in reptile and bird species (Sauropsida, Table S3 and Figure 1) showed a surprising finding: some branches appear to have lost *PAX4* and *PAX8*. It was recently suggested [44] that *PAX8* gene was lost after turtles split from other reptiles and birds, which most likely occurred ∼240 million years ago [45]–[47]. We found *PAX8* in 2 of the 3 turtle species studied (*Chrysemys picta bellii* and *Pelodiscus sinensis*; Table S3). We also found 9 *PAX* genes, including a *PAX8* segment, in a snake (*Python molurus*). The unresolved Sauropsida phylogeny (Figure 1; [48]) raises the question as to whether the loss of *PAX4* and *PAX8* is an ancestral event, or whether these losses occurred independently in distinct reptile and bird lineages.

Overall, the searches showed that all 9 *PAX* genes appear to be present in the 74 mammalian species studied (Figure 1). Although some exceptions to this general pattern were found, they are likely a consequence of the low coverage of the genome in question (e.g. *Dipodomys ordii* (kangaroo rat), which had only a 2× coverage), or due to bias toward the most popular genes, rather than a reflection of actual gene loss.

### Shared Synteny and/or Conserved Neighborhood Analysis

We performed an analysis of shared synteny (genes in the same chromosome) and/or conserved neighborhood (genes side-by-side in the same order) for all 4 *PAX* subfamilies: (1) *PAX1 a*nd *PAX9*; *(*2) *PAX2, PAX5*, *a*nd *PAX8*; (3) *PAX3 a*nd *PAX7*; (4) *PAX4 a*nd *PAX6* [7], [21]. The most conserved and similar blocks were those in which *PAX1* and *PAX9* were inserted. The others presented distinct levels of neighboring and conserved synteny.

This analysis was also used as additional evidence for the absence of *PAX4* and *PAX8*, as well as for evidence of *PAX6* duplication in some taxa, as described in the previous section.

**Figure 2:**
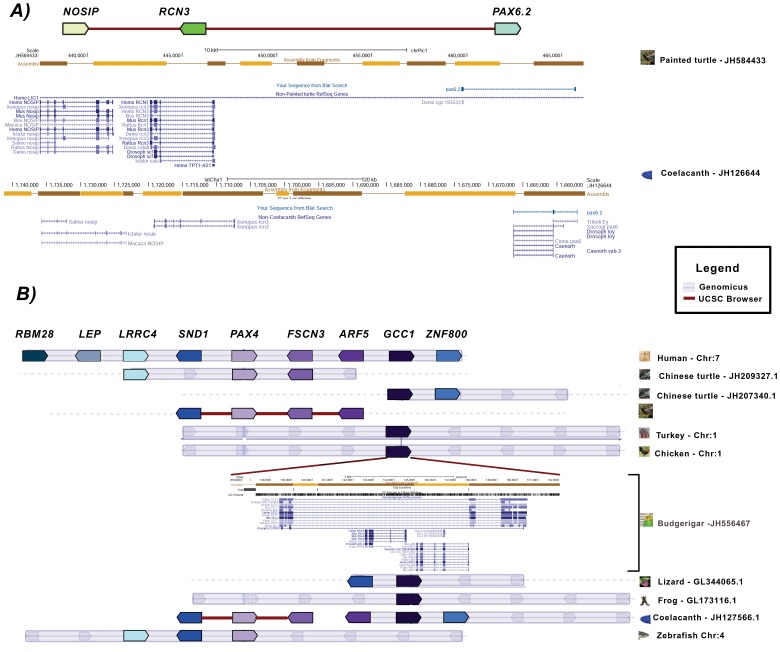
Synteny and neighborhood status for the *PAX4* and *PAX6* genes.

By using a similar approach, Ravi et al. [38] recently found that *PAX6.2* was located in close proximity to the *RCN3* and *NOSIP* genes in the elephant shark, lizard, zebrafish, and *Xenopus.* However, no *PAX6*-duplicated ortholog was found in the proximity of *NOSIP-RCN3* or elsewhere in the genomes of birds or mammals. Our analysis confirmed the presence of *PAX6.2* in *RCN3-NOSIP* in *Xenopus* and in a lizard species *(Anolis carolinensis*). Additionally, we found the *PAX6.2* gene in this same region in the coelacanth and in the painted turtle (*Chrysemys picta bellii;* Figure 2A), but failed to find the *PAX6.2-RCN3-NOSIP* block in the genomes of another turtle species (*Pelodiscus sinensis*) or in mammals and birds. Consequently, our data support the proposal that the duplication that gave rise to the *PAX6.2* gene must have occurred before the split between cartilaginous and bony fish, and that this duplication was followed by multiple independent *PAX6.2* gene losses in distinct vertebrate lineages.

We found a possible fragment of the *PAX4* gene in association with the *ARF5* gene in the budgerigar (*Melopsittacus undulatus*) genome, which was in the intronic region of the *GCC1* gene. In other vertebrate genomes, the *GCC1-ARF5-FSCN3-PAX4* block formed a conserved neighborhood (Figure 2B). Although the *PAX4* segment appeared to contain a complete paired domain, 3 independent approaches failed to predict a full functional protein.

The syntenic analysis of *PAX8* showed that the block in which it is inserted in mammals and fish is relatively well conserved in birds and reptiles, whose some genomes lack *PAX8*, providing supporting evidence for its loss.

Based on the presence of putatively nonfunctional relics (*PAX4* and *PAX8* segments in *Melopsittacus undulatus* and *Python molurus,* respectively), along with the other findings presented above, we can suggest that the loss of *PAX4* and/or *PAX8* occurred multiple times in tetrapod lineages, which also appears to be the case for *PAX6.2*. This hypothesis is compatible with findings of previous studies [49]–[56].

### Comparative Analysis of PAX Proteins

**Figure 3:**
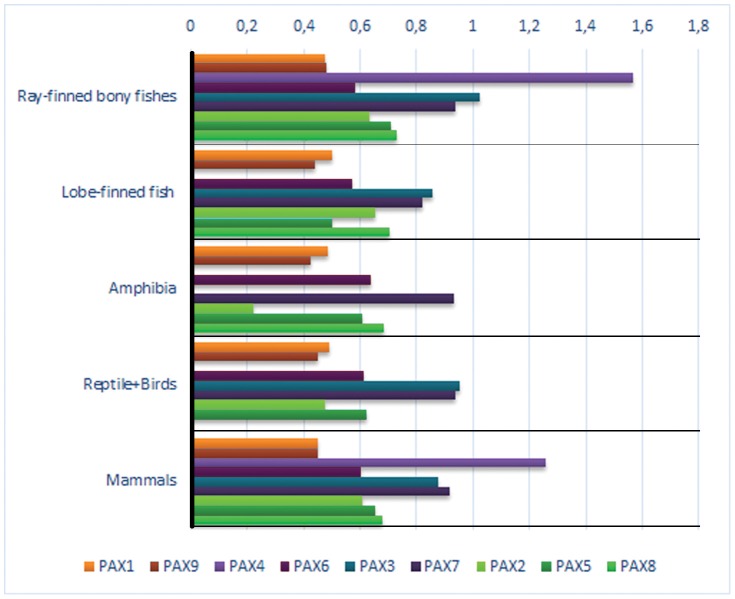
Pairwise PAX protein changes observed in different vertebrate taxa compared to that of the sea squirt.

The PAX protein analysis was performed using the 53 species whose available sequences met our quality criteria (see Material and Methods). Pairwise amino acid distances were calculated between each PAX subfamily member and the PAX protein type of its probable outgroup (the urochordata *Ciona intestinalis*) (Table S4 and Figure 3). The distances of each protein from that of its probable outgroup were also compared among the 4 subfamilies ((1) PAX1 and PAX9; (2) PAX2, PAX5, and PAX8; (3) PAX3 and PAX7; (4) PAX4 and PAX6). For instance, the distance values of the human PAX1 and PAX9 to the PAX1/9 type from *Ciona intestinalis* were 0.447 and 0.455, respectively, which was not statistically significant (*p = *0.706).

Considering all comparisons (53 chordate species and 9 PAX proteins), some specific and general patterns emerged: the distance values between PAX1 and PAX9 paralogs to their putative PAX1/9 protein did not show significant differences (*p = *0.069). In other words, the distance of PAX1 to the *Ciona Intestinalis* PAX1/9 is the same as the distance to its paralogous gene, PAX9. The same result was found when PAX3 and PAX7 were compared with PAX3/7 (*p* = 0.704).

On the other hand, the PAX4 protein was more dissimilar to its putative outgroup (PAX4/6) than it was to PAX6 (*p = *0.001). Therefore, this suggests that *PAX4* most likely emerged in 2 R together with *PAX6*. Possibly higher evolutionary rates (inferred by ω = dN/dS) would further confirm this result. A recent study indicated a possible loss of PAX4 expression from the brain in vertebrates, probably after 2 RWGD [43], may have led to relaxed constraints on gene conservation, as suggested by a higher rate of sequence divergence.

When PAX2, PAX5, and PAX8 were compared with their putative PAX 2/5/8 ancestral type, other suggestive patterns appeared. The distance between PAX8 and its ancestor was significantly different from those of the others (*p = *0.001), whereas the PAX2 and PAX5 distances did not present a significant difference (*p = *0.091).

These results could indicate that a round of complete vertebrate genome duplication most likely involved *PAX2* and *PAX5* ancestor, whereas *PAX8* emerged through local gene duplication. This would support the idea that *PAX8* is the most recent gene to appear by duplication in this family. An alternative scenario to evolution of the *PAX2/5/8* subfamily is that after 1 RWGD, two copies of the subfamily genes emerged, one resembling *PAX2/5* and the other *PAX8*. A second duplication event (2 WRGD), resulted in 4 copies of the *PAX2/5/8* subfamily, followed by loss of one of the *PAX8* duplicates. The result is the presence of *PAX2, PAX5, and PAX8* genes in jawed vertebrates [19], [20]. The relaxation of selective pressure immediately after this last partial/total duplication would be expected, which could explain the higher variation observed in *PAX8* relative to its outgroup (*PAX2/5/8*). The higher evolutionary rate (inferred by ω = dN/dS; see Material and Methods and next section) could be an alternative explanation; however, the 2 above mentioned possibilities, relative to *PAX8,* are not mutually exclusive. Redundancy in the expression of these genes likely played a central role in the loss and/or higher divergence rate of *PAX8*. In mammals, *PAX8* is mainly expressed in the kidney, ear, and thyroid gland during development, whereas *PAX2* is expressed not only in these organs and tissues, but also in others, such as the eye, pharyngeal arches, and brain [2], [3], [5], [7], [19], [20], [44]. Amphioxus (here considered as an outgroup) contains only *PAX2/5/8* and shows pleiotropic expression in most organs and tissues, implying that *PAX2, PAX5*, and *PAX8* have retained most of their ancestral expression patterns [19], [20], [44].

### Selection on *PAX* Genes

Our molecular evolution analyses (Table 1) revealed ω values <1, indicating that purifying selection has acted on *PAX* over the majority of the evolutionary history of vertebrates. This result is consistent with the idea that developmental genes are under functional restriction in metazoa [56]. The clade model D [57] performed better in a likelihood ratio test (LTR; *p*<0.001; Table 1) when compared with the neutral M1a model. This result indicates that ω values obtained for the 4 *PAX* subfamilies can vary between branches, predicting distinct molecular evolutionary patterns.

**Table 1:** Branch-site clade model D values for ω (dN/dS ratio) estimated for 2 site classes.

*PAX* subfamily	Clade Model D			M1a - Parameter estimates	P* (LRT)
	Proportion (p)	Branch type 0 (ω)	Branch type 1 (ω)		M1a X Clade Model
		*PAX9*	*PAX1*	*PAX1/PAX9*	
*PAX1* and *PAX9*	0.87268	0.00186	0.00186	p: 0.98611 0.01389	<0.001
	0.12732	0.08140	0.09185	ω: 0.01023 1.00000	
		PAX2/PAX5	PAX8	*PAX2/PAX5/PAX8*	
*PAX2, PAX5*, and *PAX8*	0.62462	0.01192	0.01192	p: 0.99999 0.00001	<0.001
	0.37538	0.00027	0.00117	ω: 0.00650 1.00000	
		*PAX3*	*PAX7*	*PAX3/PAX7*	
*PAX3* and *PAX7*	0.68577	0.01016	0.01016	p: 0.99360 0.00640	<0.001
	0.31423	0.09976	0.18923	ω: 0.03997 1.00000	
		*PAX4*	*PAX6*	*PAX4/PAX6*	
*PAX4* and *PAX6*	0.61493	0.02335	0.02335	p: 0.83385 0.16615	<0.001
	0.38507	0.30208	0.01784	ω: 0.06400 1.00000	

* Degrees of freedom: 2; LRT: 2Δl = 2(l 1− l 0);

Although all estimated ω values were less than 1, which suggested the action of negative selection, a possible relaxation of this selective constraint was revealed when the subfamilies were compared. In 38% of the sites, the *PAX4* ω value was 16 times greater than that of the *PAX6* ω value. Additionally, in 37% and 31% of the sites, the *PAX8* and *PAX7* ω values were approximately 2 and 4 times greater than the *PAX2-PAX5* and *PAX3* ω values, respectively (Table 1). These results suggest that *PAX4, PAX8*, and *PAX7* have experienced relatively more modifications than the other *PAX* genes.

### Gene Phylogeny Analysis

Bayesian Monte Carlo Markov Chain trees were built from 2 *PAX* subfamily data sets (*PAX2, PAX5*, and *PAX8; PAX4* and *PAX6*), in which genes were lost in some lineages, and presented greater molecular evolutionary rates wherever they were not lost. Well-defined clusters were observed that separated the 3 and 2 genes of each subfamily, respectively (Figure S1 illustrates the *PAX2, PAX5*, and *PAX8* tree). These results indicate again the conservative nature of purifying selection that has driven molecular evolution of the *PAX* gene. As expected, the *PAX* genes found in the tunicate *Ciona intestinalis* (*PAX2/5/8* and *PAX4/6*) lead to basal branches in Figure S1 in the other subfamily (data not shown). In some cases, recovery of the class phylogeny of the species is apparent as clear mammal, fish, and bird clades can be observed in the *PAX2* cluster. Similar topologies and statistical robustness were obtained using the maximum likelihood method (data not shown). The trees, however, do not provide additional evidence about the differences in evolutionary rates of genes observed within each subfamily.

### Conclusion

Overall, purifying selection appears to be the main factor responsible for molecular evolution of the *PAX* family in chordate species. However, there are some indications of potential group-specific changes that are beyond this general pattern. There was a loss of *PAX4* and *PAX8* in lizards and birds. Accelerated evolutionary rates were suggested for the *PAX4, PAX8* and *PAX7* genes. The accumulation of variation (at least in some sites), due to an initial relaxation of purifying selection, may indicate the beginning of a process that enabled evolvability of the system.

Results of the present study revealed that some *PAX* genes experienced striking changes in the course of their evolutionary trajectory, which emphasizes the point that even developmental master genes might not follow universal patterns of molecular evolution. Functional retention and loss, subfunctionalization, as well as neofunctionalization can also be observed in developmental genes.

The asymmetric evolution of the *PAX* family genes observed here, as evidenced by uneven events of duplications and deletions are compatible with the emergence of adaptive novelties during chordate radiation.

## Materials and Methods

### Data Collection

Nucleotide and amino acid sequences for all available *PAX* genes in chordate species were obtained using Biomart (Ensembl v66–70 - http://www.ensembl.org/biomart/martview/; [58], [59]). The Protein Domains/Limit filter (InterPro (ID): IPR00152) was used as a parameter to identify the *PAX* genes or the paired box domains. A second approach was the inspection of one-to-one ortholog gene maps, which were also obtained from Biomart.

BLAST/BLAT searches in Ensembl (http://www.ensembl.org/Multi/blastview), UCSC databases (http://genome.ucsc.edu/), and in the NCBI Genebank (genomic BLAST http://www.ncbi.nlm.nih.gov/sutils/genom_table.cgi?organism=8496&database=8496) were also conducted in order to identify possible unannotated orthologs. The Pre!Ensembl database (http://pre.ensembl.org/index.html) was used to access the new draft released genomes. Finally, we applied hidden Markov models (HMMER) web service searching sequence databases for homologs of *PAX* amino acid sequences in the NR and Uniprot collections [60].

The genomic sequences of possible unannotated orthologs were verified using three programs that can predict open reading frames (ORF): BESTORF (http://linux1.softberry.com/berry.phtml?topic=bestorf&group=programs&subgroup=gfind), GeneWise (http://www.ebi.ac.uk/Tools/psa/genewise/) [61], and STAR ORF (http://star.mit.edu/orf/index.html).

The following procedures were also adopted: (a) for genes encoding multiple transcripts, the transcript with the longest genomic transcribed length was selected; (b) a high identity (up to 70%) with the paired domain was accepted as indicating a *PAX* family member; (c) possible gene losses were accepted only when they were observed in multiple species as well as in high coverage genome assemblies; (d) a subset of 53 species was selected for the evolutionary analyses, since their sequences had the best alignment, and they were optimized for analysis with a higher number of sites.

### *PAX* Gene Family Synteny and Neighborhood Status

Mapping adjacent genes into *PAX* synteny regions was achieved with the Genomicus website v70.01 (http://www.dyogen.ens.fr/genomicus-70.01/cgi-bin/search.pl [62]). Additionally, we manually searched the Ensembl and UCSC genome browsers for the same purpose.

### Variation in the *PAX* Family

The amino acid sequences were aligned using the MUSCLE algorithm [63] included in Mega (version 5.0) [64], which were verified with the GUIDANCE web service using the MAFFT algorithm [65]. Mega (version 5.0) software was employed to evaluate variability in the *PAX* groups using the pairwise distance of members of each subfamily from the gene of its probable outgroup, the tunicate (*Ciona intestinalis)*. SPSS (version 16) software was used to calculate the statistical significance of differences between *PAX1/9*, *PAX2/5/8*, *PAX3/7*, and *PAX4/6* paralogous sequences using the paired Student’s *t-*test.

### Tests for Selection

Patterns of selection and rates of evolutionary changes in the *PAX* family were evaluated using standard tests [66]–[68]. We used the phylogeny-based maximum likelihood analysis of ω (*dN*/*dS*) as implemented in the CODEML program of the PAML 4.4 package to statistically test for positive selection and/or relaxation of functional constraints. The heterogeneity of evolutionary rates among paralogous groups was tested using the CODEML program in PAML4.4 clade models [57]. Branches on the phylogeny were divided into 2 clades *a priori*, and a likelihood ratio test (LRT) was used to evaluate divergences in selective pressures between them, as indicated by different ω ratios. We employed the clade model type D that assumes 2 site classes, which was compared with the neutral model M1a by an LRT with 2 degrees of freedom. This comparison was primarily used to detect positive selection, but our goal here was also to evaluate acceleration during the evolutionary history through direct inferences of *dN/dS* differences.

Empirical Bayes approaches, implemented in CODEML, were also used to infer which of the *PAX* sequences sites might have evolved under positive selection. To determine sites under selection, the naive-empirical Bayes (NEB) test was employed. The unrooted tree input file for PAML4.4 analyses was a phylogenetic tree provided by Ensembl, which was edited using PhyloWidget for the 53 species included in this study.

### Gene Phylogeny

Data from *PAX4* and *PAX6,* as well as from *PAX2, PAX5*, and *PAX8* gene subfamilies were used to construct phylogenetic trees. The comparison was performed using a mixed Bayesian Monte Carlo Markov Chain sampler for phylogenetic reconstruction using protein alignments in PhyloBayes [69] on the web server Bioportal from the University of Oslo. Additionally, we built trees using the maximum likelihood method (Mega, version 5.0 [64]) using the same dataset.

## Supporting Information

Figure S1 ***PAX2, PAX5*** **,** ***and PAX8*** **gene subfamilies phylogeny based on the Bayesian Monte Markov Chain method.**
(JPG)

Table S1 **Description and functions of** ***PAX*** **genes.**
(DOCX)

Table S2 **Species considered in this study and font of hits.**
(XLSX)

Table S3 **Presence of PAX genes in 188 Chordate species.**
(XLSX)

Table S4 **Pairwise distances between the** ***PAX*** **subfamilies and the outgroup** ***PAX*** **sea squirt.**
(DOCX)
