# Supplementary figures and images for: Evolutionary History of Chordate PAX Genes: Dynamics of Change in a Complex Gene Family
Source: PLoS One. 2013 Sep 2;8(9):e73560. doi: 10.1371/journal.pone.0073560 (PMC3759438; doi:10.1371/journal.pone.0073560)

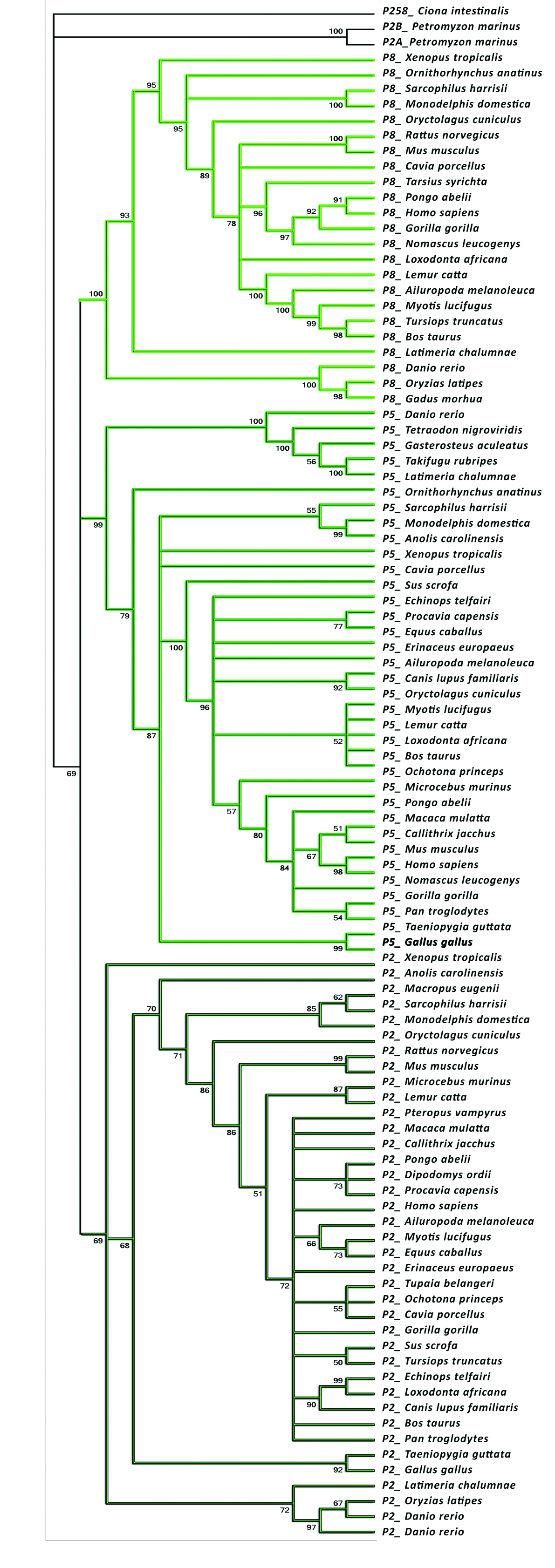

Supplement: Figure S1 — PAX2, PAX5 , and PAX8 gene subfamilies phylogeny based on the Bayesian Monte Markov Chain method. (JPG) [file pone.0073560.s001.jpg]
