# Supplementary material for: Evolutionary History of Chordate PAX Genes: Dynamics of Change in a Complex Gene Family
Source: PLoS One. 2013 Sep 2;8(9):e73560. doi: 10.1371/journal.pone.0073560 (PMC3759438; doi:10.1371/journal.pone.0073560)
Supplement: Table S1 — Description and functions of PAX genes. (DOCX) [file pone.0073560.s002.docx]

**Table S1.** Description and functions of *PAX* genes

| **Gene** | **Description** | **Summary** | **Function** | **Human Disorders** |
| --- | --- | --- | --- | --- |
| *PAX1* | paired box 1 | The PAX genes, including PAX1, are a highly conserved family of developmental control genes that encode transcription factors and have been shown to play a role in pattern formation during embryogenesis in vertebrates. | This protein is a transcriptional activator. Play a role in the formation of segmented structures of the embryo and have a important role in the normal development of the vertebral column. | Vertebral anomalies. |
| *PAX2* | paired box 2 | PAX2 encodes paired box gene 2, one of many human homologues of the Drosophila melanogaster gene *prd*. The central feature of this transcription factor gene family is the conserved DNA-binding paired box domain. PAX2 is believed to be a target of transcriptional supression by the tumor supressor gene WT1. Alternative splicing of this gene results in multiple transcript variants. | Probable transcription factor that may have a role in kidney cell differentiation. Has a critical role in the development of the urogenital tract, the eyes, and the CNS. | Defects in PAX2 are the cause of renal-coloboma syndrome (RCS); also known as papillorenal syndrome or optic nerve coloboma with renal disease. RCS is an autosomal dominant disease characterized by the association of renal hypoplasia, vesicoureteral reflux and dysplasia of the retina and optic disk. |
| *PAX3* | paired box 3 | This gene is a member of the paired box (PAX) family of transcription factors. These genes play critical roles during fetal development. Alternative splicing results in transcripts encoding isoforms with different C-termini. | Transcription factor have a role in myogenesis, and in CNS. | Mutations in paired box gene 3 are associated with Waardenburg syndrome, craniofacial-deafness-hand syndrome, and alveolar rhabdomyosarcoma. The translocation t(2;13)(q35;q14), which represents a fusion between PAX3 and the forkhead gene, is a frequent finding in alveolar rhabdomyosarcoma. |
| *PAX4* | paired box 4 | This gene is a member of the paired box (PAX) family of transcription factors. These genes play critical roles during fetal development and cancer growth. The paired box 4 gene is involved in pancreatic islet development and mouse studies have demonstrated a role for this gene in differentiation of insulin-producing beta cells. | Plays an important role in the differentiation and development of pancreatic islet beta cells. Transcriptional repressor that binds to a common element in the glucagon, insulin and somatostatin promoters. Competes with PAX6 for this same promoter binding site. | Defects in PAX4 are a cause of noninsulin-dependent diabetes mellitus (NIDDM) also known as diabetes mellitus type 2 or maturity-onset diabetes. |
| *PAX5* | paired box 5 | This gene encodes a member of the paired box (PAX) family of transcription factors. The central feature of this gene family is a novel, highly conserved DNA-binding motif, known as the paired box. PAX proteins are important regulators in early development, and alterations in the expression of their genes are thought to contribute to neoplastic transformation. This gene encodes the B-cell lineage specific activator protein that is expressed at early, but not late stages of B-cell differentiation. Its expression has also been detected in developing CNS and testis and so the encoded protein may also play a role in neural development and spermatogenesis. | May play an important role in B-cell differentiation as well as neural development and spermatogenesis. Involved in the regulation of the CD19 gene, a B-lymphoid-specific target gene | A chromosomal aberration involving PAX5 is a cause of acute lymphoblastic leukemia. Translocation t(9;18)(p13;q11.2) with ZNF521. Translocation t(9;3)(p13;p14.1) with FOXP1. Translocation t(9;12)(p13;p13) with ETV6 |

**Table S1.** Cont.

| **Gene** | **Description** | **Summary** | **Function** | **Human Disorders** |
| --- | --- | --- | --- | --- |
| *PAX6* | paired box 6 | This gene encodes paired box gene 6, one of many human homologs of the Drosophila melanogaster gene prd. In addition to the hallmark feature of this gene family, a conserved paired box domain, the encoded protein also contains a homeo box domain. Both domains are known to bind DNA, and function as regulators of gene transcription. This gene is expressed in the developing nervous system, and in developing eyes. Alternatively spliced transcript variants encoding either the same or different isoform have been found for this gene. | Transcription factor with important functions in the development of the eye, nose, central nervous system and pancreas. Required for the differentiation of pancreatic islet alpha cells . Competes with PAX4 in binding to a common element in the glucagon, insulin and somatostatin promoters. Regulates specification of the ventral neuron subtypes by establishing the correct progenitor domains . | Mutations in this gene are known to cause ocular disorders such as aniridia and Peter's anomaly. |
| *PAX7* | paired box 7 | This gene is a member of the paired box (PAX) family of transcription factors. Three transcript variants encoding different isoforms have been found for this gene. | Play a role in myogenesis, satelite cells an CNS. | Defects in PAX7 are a cause of rhabdomyosarcoma type 2 (RMS2). It is a form of rhabdomyosarcoma, a highly malignant tumor of striated muscle derived from primitive mesenchimal cells and exhibiting differentiation along rhabdomyoblastic lines. Rhabdomyosarcoma is one of the most frequently occurring soft tissue sarcomas and the most common in children. |
| *PAX8* | paired box 8 | This gene encodes a member of the paired box (PAX) family of transcription factors. This nuclear protein is involved in thyroid follicular cell development and expression of thyroid-specific genes. Alternatively spliced transcript variants encoding different isoforms have been described. | Transcription factor for the thyroid-specific expression of the genes exclusively expressed in the thyroid cell type, maintaining the functional differentiation of such cells | Mutations in this gene have been associated with thyroid dysgenesis, thyroid follicular carcinomas and atypical follicular thyroid adenomas. |
| *PAX9* | paired box 9 | This gene is a member of the paired box (PAX) family of transcription factors. The specific function of the paired box 9 gene is unknown but it involve development of stratified squamous epithelia as well as various organs and skeletal elements. | Transcription factor required for normal development of thymus, parathyroid glands, ultimobranchial bodies, teeth, skeletal elements of skull and larynx as well as distal limbs. | Defects in PAX9 are the cause of tooth agenesis selective type 3 (STHAG3) . A form of selective tooth agenesis, a common anomaly characterized by the congenital absence of one or more teeth. |

Data collected from Ensemble (http://www.ensembl.org/); OMIM (http://www.ncbi.nlm.nih.gov/sites/entrez?db=omim); Uniprot (http://beta.uniprot.org/); Genecards (http://www.genecards.org/index.shtml);
