# Supplementary material for: Evolutionary History of Chordate PAX Genes: Dynamics of Change in a Complex Gene Family
Source: PLoS One. 2013 Sep 2;8(9):e73560. doi: 10.1371/journal.pone.0073560 (PMC3759438; doi:10.1371/journal.pone.0073560)
Supplement: Table S4 — Pairwise distances between the PAX subfamilies and the outgroup PAX sea squirt. (DOCX) [file pone.0073560.s005.docx]

**Table S4.** Pairwise distances between the *PAX* subfamilies and the outgroup *PAX* sea squirt ^1^.

| **Species** | ***PAX1*** | ***PAX9*** | ***PAX4*** | ***PAX6*** | ***PAX3*** | ***PAX7*** | ***PAX2*** | ***PAX5*** | ***PAX8*** |
| --- | --- | --- | --- | --- | --- | --- | --- | --- | --- |
| Lamprey | 0.398 | 0.398 | * | * | * | * | 0.660 | * | * |
| Zebrafish | 0.438 | 0.468 | 1.569 | 0.578 | 0.946 | 0.945 | 0.634 | 0.646 | 0.737 |
| Cod | 0.502 | 0.461 | MD | 0.584 | 1.249 | 0.928 | MD | MD | 0.732 |
| Medaka | 0.463 | MD | MD | 0.581 | 1.005 | 0.904 | 0.634 | 0.705 | 0.717 |
| Stickleback | 0.507 | 0.503 | ND | 0.581 | 1.002 | 0.944 | MD | 0.676 | MD |
| Fugu | 0.471 | 0.488 | MD | 0.585 | 1.024 | 0.932 | MD | 0.796 | MD |
| Tetraodon | 0.489 | 0.485 | MD | 0.571 | 0.927 | 0.959 | MD | 0.717 | MD |
| Coelacanth | 0.502 | 0.442 | MD | 0.573 | 0.857 | 0.819 | 0.651 | 0.502 | 0.707 |
| Frog | 0.486 | 0.425 | MD | 0.639 | MD | 0.932 | 0.222 | 0.608 | 0.685 |
| Lizard | 0.490 | 0.467 | **L** | 0.579 | 0.859 | MD | 0.210 | 0.605 | L |
| Zebra Finch | 0.522 | 0.470 | **L** | 0.675 | 0.855 | MD | 0.607 | 0.627 | L |
| Turkey | 0.458 | 0.436 | **L** | MD | 1.142 | 0.939 | MD | MD | L |
| Chicken | 0.486 | 0.435 | **L** | 0.581 | MD | 0.932 | 0.606 | 0.639 | L |
| Platypus | 0.445 | 0.422 | 1.018 | 0.833 | 0.862 | MD | MD | 0.606 | 0.690 |
| Tasmanian devil | 0.429 | 0.425 | 1.155 | 0.576 | MD | 0.924 | 0.607 | 0.623 | 0.664 |
| Wallaby | 0.429 | 0.428 | MD | 0.669 | 0.864 | MD | 0.610 | MD | MD |
| Opossum | 0.429 | 0.428 | MD | MD | 0.876 | MD | 0.610 | 0.612 | 0.685 |
| Armadillo | MD | 0.443 | MD | MD | MD | MD | MD | MD | MD |
| Tenrec | MD | 0.460 | MD | MD | MD | MD | 0.615 | 0.610 | MD |
| Hyrax | 0.457 | MD | 1.347 | 0.579 | 0.871 | MD | 0.606 | 0.611 | MD |
| Elephant | 0.462 | 0.441 | 1.440 | 0.578 | 0.869 | 0.907 | 0.610 | 0.629 | 0.670 |
| Shrew | MD | 0.451 | 1.351 | MD | MD | MD | MD | MD | MD |
| Hedgehog | 0.476 | MD | 1.237 | MD | MD | MD | 0.625 | 0.626 | MD |
| Megabat | 0.477 | MD | 1.268 | MD | MD | MD | 0.610 | MD | MD |
| Microbat | 0.465 | 0.455 | 1.504 | 0.578 | 0.878 | MD | 0.646 | 0.620 | 0.677 |
| Dog | 0.471 | 0.455 | 1.212 | MD | 0.866 | 0.909 | 0.610 | 0.620 | MD |
| Panda | 0.461 | 0.455 | 1.235 | 0.578 | 0.881 | 0.904 | 0.610 | 0.620 | 0.677 |
| Horse | MD | 0.453 | 1.197 | 0.641 | 0.884 | MD | 0.610 | 0.631 | MD |
| Cow | 0.470 | 0.455 | 1.270 | 0.578 | 0.876 | 0.911 | 0.610 | 0.620 | 0.667 |
| Dolphin | 0.470 | MD | 1.373 | MD | MD | 0.923 | 0.610 |  | 0.696 |
| Pig | 0.470 | 0.455 | 1.191 | 0.578 | MD | MD | 0.599 | 0.620 | MD |
| Pika | MD | MD | 1.318 | 0.581 | MD | 0.952 | 0.610 | 0.620 | MD |
| Rabbit | 0.460 | 0.508 | 1.207 | 0.578 | 0.876 | 0.916 | 0.620 | 0.626 | 0.676 |
| Squirrel | MD | MD | 1.299 | MD | MD | MD | MD | MD | MD |
| Guinea Pig | 0.448 | 0.449 | 1.160 | 0.577 | 0.875 | MD | 0.607 | 0.620 | 0.667 |
| Kangaroo rat | 0.334 | MD | 1.265 | MD | MD | 0.923 | 0.594 | MD | MD |
| Rat | 0.453 | 0.465 | 1.429 | 0.578 | 0.878 | 0.912 | 0.626 | MD | 0.674 |
| Mouse | 0.454 | 0.465 | 1.277 | 0.578 | 0.872 | 0.914 | 0.626 | 0.620 | 0.672 |
| Tree Shrew | MD | 0.455 | MD | MD | 0.873 | MD | 0.459 | MD | MD |
| Bushbaby | 0.459 | 0.437 | 1.240 | 0.578 | 0.874 | MD | 0.610 | 0.620 | 0.677 |
| Mouse Lemur | 0.452 | 0.455 | 1.324 | 0.578 | 0.882 | 0.930 | 0.588 | 0.620 | MD |
| Marmoset | 0.448 | 0.456 | 1.218 | 0.578 | 0.883 | 0.905 | 0.610 | 0.620 | MD |
| Macaque | 0.447 | 0.460 | 1.261 | 0.578 | 0.876 | 0.932 | 0.610 | 1.490 | MD |
| Gibbon | 0.447 | 0.455 | 1.231 | 0.578 | 0.876 | 0.919 | MD | 0.620 | 0.679 |
| Orangutan | 0.447 | 0.455 | 1.204 | MD | 0.876 | 0.910 | 0.610 | 0.620 | 0.683 |
| Gorilla | 0.447 | 0.455 | 1.219 | 0.578 | 0.876 | 0.937 | 0.610 | 0.620 | 0.679 |
| Chimpanzee | 0.447 | 0.455 | 1.172 | 0.772 | 0.876 | 0.921 | 0.610 | 0.620 | MD |
| Human | 0.447 | 0.455 | 1.180 | 0.578 | 0.876 | 0.921 | 0.610 | 0.620 | 0.679 |

^1^MD = missing data - the sequences are too short or too divergent to be aligned;* presence of the *PAX* putative outgroup; L= genes losses.
